# Delayed decision-making, failed abortion attempts and factors associated with second-trimester abortions in Liberia

**DOI:** 10.1186/s12978-025-02102-1

**Published:** 2025-11-04

**Authors:** George B. Davis, Kenneth Juma, Stephanie Kung, Laura A. Skrip

**Affiliations:** 1 School of Public Health, College of Health Sciences, University of Liberia, Monrovia, 1000-10 Liberia; 2 Quantitative-Data for Decision-Making (Q4D) Lab, Monrovia, Liberia; 3 African Population and Health Research Center (APHRC), Nairobi, Kenya; 4 Guttmacher Institute, New York, USA

**Keywords:** Second trimester abortion, Liberia, Legally restrictive settings, Post-abortion care

## Abstract

**Background:**

Abortion is legally restricted in Liberia with few exceptions. The country also has among the highest maternal mortality ratios in the world, with an estimated 742 deaths per 100,000 live births. The contributions of unsafe abortion to maternal mortality have been investigated in Liberia, but less is known about the magnitude of and circumstances around abortions occurring in the second trimester. We aimed to assess the prevalence and factors associated with second trimester induced abortions among women seeking post-abortion care in Liberia.

**Methods:**

We conducted a secondary analysis of data from a Prospective Morbidity Survey conducted at health facilities across Liberia from October to December 2021. We analyzed data from women seeking post-abortion care (PAC) for induced abortions to calculate the proportion occurring in the second trimester and to identify factors associated with second-trimester termination.

**Results:**

Among women seeking PAC after induced abortion, 35 out of 185, or 19.0% (95% CI: 14.0%, 25.3%), had induced abortions during the second trimester. This represented just over 8% (95% CI: 5.9%, 11.0%) of all women seeking PAC. The distribution of women across age, employment status, religious affiliation, and relationship status was largely similar between those who induced abortion in the second trimester and those who terminated their pregnancies in the first trimester. They also had similar reproductive histories, with no differences in history of spontaneous or induced abortion or in the number of living children. About 68% of women who ultimately terminated their pregnancies in the second trimester reported previous attempts at inducing abortion for the same pregnancy. Compared to women with secondary or tertiary level education, women with no formal education or primary school education had 3.1 times the odds of second-trimester abortion (OR = 3.1, 95% CI: 1.4-7.0, *P* = 0.006). Likewise, women who reported that their pregnancies were intended had 2.2 times the odds of a second-trimester abortion compared to women reporting unintended pregnancies (OR = 2.2, 95% CI: 1.0-4.8, *P* = 0.045). The direct effects of these factors exhibited some degree of mediation by having made one or more previous attempts at termination and/or by making the decision to terminate later in the pregnancy.

**Conclusions:**

Second-trimester abortion constitutes nearly one-fifth of induced abortions for which PAC is being sought in Liberia. The high prevalence of repeat abortion attempts for the same pregnancy points to limitations in access to safe and effective abortion methods. Similarly, the high rates of unintended pregnancy in the study sample despite reported use of family planning suggests gaps in the quality of family planning services and commodities accessible to women. More comprehensive sexual and reproductive health education is warranted.

## Abstract (plain language summary)

This study focused on second-trimester abortions in Liberia, where induced abortion is highly restricted by law but still prevalent. The researchers analyzed data from interviews with women seeking post-abortion medical care. Approximately 19% of women who reported or were assessed as having intentionally terminated their pregnancies had induced more than 12 weeks after getting pregnant. Many of these women had attempted to end their pregnancies before but were only successful in the second trimester. Factors such as lower education and having intended for the pregnancy were more common among women having second-trimester abortions compared to women terminating in the first trimester. The findings highlight the need for improved access to safe and effective abortion methods and family planning services, as well as enhanced education on sexual and reproductive health.

## Background

In Sub-Saharan Africa (SSA), over 90% of women aged 15–49 years live in countries where induced abortion is entirely prohibited or otherwise restricted to serious medical cases that threaten a woman’s life [1]. Regardless of the degree of legal restriction, abortion incidence has been relatively uniform across time and space in SSA, suggesting that legal restrictions barely reduce the propensity towards abortion, but rather drive women to unsafe abortion methods and procedures [2]. The SSA region has an estimated abortion rate of 33 induced abortions per 1000 women of reproductive age as of 2019 [1, 3]. Although there is no evidence that the rate of abortion has changed in recent years, the absolute number of abortions is increasing rapidly with population growth, and this has consequences for the magnitude of maternal death and disability associated with unsafe abortion-related complications [1].

An estimated 45% of all abortions globally are considered unsafe, while more than 75% of abortions occurring in SSA are unsafe [3–5]. Complications related to unsafe abortion account for at least 7-13% of maternal deaths in SSA, estimated at 37 deaths per 100,000 live births annually [2, 5, 6]. The approximately 10–15% of unsafe abortions estimated to take place in the second trimester pose the greatest risk of maternal mortality and serious complications such as hemorrhage, infection, and uterine rupture and perforation [7, 8]. This is particularly the case in low-resource nations where access to safe surgical procedures, most often required for later term abortions, is limited [1, 9].

Existing literature has found that termination of pregnancies in the second trimester is mainly linked to factors such as late recognition of pregnancy, delayed referrals when complications arise, financial barriers, or lack of awareness on options available and on the health consequences of delayed care-seeking [10–13]. Other factors include limited access to abortion services earlier in the pregnancy, especially in legally restrictive settings but even in settings where the procedure is legally available, due to low provider willingness to perform abortions [14–16]. Such structural factors around limited availability of or access to safe abortion services and delayed decision-making have been found to be associated with attempts to self-induce pregnancy before presenting to a health facility [17].

In the West African country of Liberia, abortion is highly legally restricted. The Liberian Penal Law—Title 26 Revised, 1978—criminalizes abortion as an offense against the family, and abortion is only permitted to save a woman’s life, protect her health, in cases of rape, incest, felonious intercourse, or serious fetal impairment, or when a pregnant woman lacks the capacity to care for her deficient physical or mental health. Two doctors must certify that a woman/girl meets these conditions and only a doctor can perform the abortion. Access to legal abortion under the allowed exceptions is extremely rare due to low knowledge of the law, fear of repercussions, and social stigma [18].

Despite legal, health system, and cultural barriers, induced abortion is common among women in Liberia: a 2021 incidence study estimated an abortion rate of 30.7 per 1,000 women of reproductive age, and estimated that 35% of all pregnancies end in abortion [19]. Maternal deaths related to unsafe abortion have been reported [20, 21], and patients seeking post-abortion care (PAC)–which is legal in Liberia–for induced abortion in the second trimester have been observed to have severe complications, such as sepsis, which accounts for 15% of all maternal deaths [20]. As Liberia has one of the highest maternal mortality ratios globally with 742 deaths per 100,000 live births [22], strengthening efforts towards reducing preventable morbidity and mortality are essential to advancing its sexual and reproductive health and rights (SRHR) agenda.

However, to date, limited empirical evidence exists around abortion in Liberia (e.g [18, 20, 21, 23]). Available studies underscore a need for expanded access to comprehensive abortion services and greater understanding of the factors around abortion-related decision-making and care-seeking in the Liberian context. More evidence specifically around abortions in the second trimester could provide actionable insights around unmet SRHR needs that may be resulting in higher risk, later-term pregnancy termination.

To begin to address this gap, we undertook a secondary analysis of data collected from a nationally representative sample of women seeking PAC in order to (1) assess the proportion of induced abortions that occur in the second trimester among women seeking post-abortion care (PAC) and (2) investigate the demographic, contextual, and reproductive health factors associated with induced abortions occurring in the second trimester. We also provide detailed information around earlier unsuccessful attempts at termination which was hypothesized to be a mediator in the causal pathway to a later term abortion.

## Methods

### Study setting

The study was conducted in Liberia, a West African country with a population of approximately 5.2 million people [24]. Liberia is geographically divided into 15 counties, which are further grouped into five geographical regions. The health system is organized into three distinct levels including clinic, health center, and hospital. Health facilities in the country can either be public facilities (government owned through the Ministry of Health), or private-for-profit, faith-based, and concession facilities (all privately owned).

### Sampling and data collection

Data from this analysis were drawn from a larger study investigating the incidence of induced abortion, unintended pregnancy, and severity of abortion-related complications in Liberia in 2021 [19]. The larger study included a Health Facility Survey (HFS), administered among a nationally representative sample of health facilities capable of providing PAC services. A detailed description of the sampling strategy is described elsewhere [19]. Briefly, the study team sampled a total of 132 facilities for the HFS, which represents 100% of hospitals that provide PAC in Liberia as of 2021, 84% of all health centers, and 7.5% of all clinics. The response rate for the survey was 97%, which corresponded to a final sample of 128 facilities.

All of the 128 health facilities in the HFS were considered for participation in the Prospective Morbidity Survey (PMS), from which the data for this analysis are derived. Participation in the PMS required that each health facility send at least one health provider (typically a nurse, midwife or a physician assistant) to attend one-week of training on the study aims and rationale, research ethics, data collection, and the study instruments. Ultimately, 78% of the HFS facilities agreed to participate in the PMS by sending a provider to attend the training, and as such, the final sample for the PMS was 100 facilities.

PMS data collection was prospective over a period of 30-days within each participating facility. All women presenting at the participating facilities during the study period seeking care and treatment for post-abortion complications and whose pregnancy gestation was 28 weeks or lower were eligible to participate in the study. Women who presented with ectopic pregnancies, molar pregnancies, or blighted ovum were excluded. Trained research assistants worked with the providers at each facility to determine the eligibility of each patient to the study.

The PMS survey had two questionnaires. The first questionnaire targeted PAC patients and gathered information on socio-demographic characteristics, reproductive histories, experiences accessing and receiving care, delays to care, and the circumstances surrounding the abortion (spontaneous or induced) that led them to seek care. The second questionnaire was administered to the patient’s health provider and gathered clinical information regarding the patient’s case. After the research assistants determined eligibility, PAC patients were taken through the informed consent process and were required to consent separately for the patient questionnaire as well as consenting to allow their provider to be interviewed using the provider questionnaire. In case the woman was incapacitated due to a severe complication or maternal death and unable to consent, the study team received approval from the ethical review board to interview the woman’s provider in order to capture her clinical details.

### Study variables

The study population included all women who sought PAC for both induced and spontaneous abortions. The determination of whether an abortion was spontaneous or induced was done in two ways. First, we used a self-reported variable - where the patient responded to a question asking *“Did you or someone else do anything to interfere with the continuation of this pregnancy?”* Second, the healthcare provider also gave their perspectives on the type of abortion by responding to a question asking whether - *“Based on your overall assessment of the client and your clinical examination findings*,* how would you classify the patient’s abortion?”*, with response options including probably induced, possibly induced, most likely spontaneous, and don’t know. Given the legal context of Liberia and the stigma that surrounds abortion, it was assumed that a woman would not report that she had induced an abortion unless that were the actual case. Therefore, self-report of induced abortion was assumed accurate; for participants who did not report the type of abortion or who reported spontaneous abortion, the provider’s perspective was also considered. The provider’s perspective on abortion type was based on a collection of findings from the patient’s clinical examination. Accordingly, if the patient self-reported induced abortion and/or if the provider indicated that the abortion was “possibly” or “probably” induced, the abortion was considered induced. When the provider indicated that the abortion was “most likely spontaneous” and the patient did not report inducing the abortion, the abortion was classified as spontaneous.

Timing of induced abortion (First versus Second trimester) was the primary outcome/dependent variable for this study. Gestational age at time of abortion was classified as first trimester (12 weeks or less) or second trimester (13 weeks or more) and was determined using self-reported whole-number weeks from the first-day of the last menstrual period, when available. For the approximately 36% (*n* = 60) of participants with missing self-reported gestational age, provider-estimated time of abortion during assessment at the PAC visit was used. Of note, there was high correlation between the self-reported and provider-estimated gestational age at abortion, when data for both variables was available.

It was hypothesized that sociodemographic and reproductive characteristics, and/or the situational experience of the current pregnancy could be associated with occurrence of induced abortion in the second-trimester. Accordingly, study variables were selected to represent those factors and experiences. The sociodemographic independent variables included age, residence in an urban or rural setting, marital status, religion, educational level, and employment status (working or not working). For reproductive characteristics,pregnancy and family planning history, number of children, gravidity, history of spontaneous abortion, history of induced abortion, and history of unintended pregnancy were considered. Variables related to the context of the current pregnancy included time to make a decision to abort (in weeks), intention to get pregnant prior to conception, and being on family planning prior to conception. Moreover, reports of prior unsuccessful attempts to end the current pregnancy were investigated as a particular variable of interest and considered to mediate the effects of other independent variables on the outcome. Given the legal restrictions around abortion in Liberia, it was assumed that self-managed attempts at abortion may be less safe—either using unrecommended methods and/or without the supervision of a trained provider—and possibly unsuccessful.

Ahead of analyses, several study variables were reformatted. For all variables, a response of “*Don’t know*” or “*Refused to disclose*” was considered missing data. Also, due to the small counts within categories for some variables, response options were collapsed. Relationship status was dichotomized into “*Living with Man*” and “*Not Living with Man*.” Religious affiliations were grouped as “*Christian*” and “*Muslim and other*.” The variable reflecting the highest level of education completed was reduced to four for the descriptive statistics and bivariable analysis. For ease of interpretation in multivariable analysis, education level was dichotomized into “*Primary or No Education*” and “*High school and Above*.” The source of income variable was used to generate an employment variable, categorizing participants as “*Not working*” or “*Working*.”

Moreover, the count variables capturing frequency of past spontaneous abortion, past induced abortion, and past unintended pregnancy were dichotomized to reflect “*Never*” for no past experience and “*One or more*” for any past experiences. The pregnancy intention variable was simplified to two levels: “*Wanted to get pregnant and give birth*” and “*Did not want to get pregnant.*” Additionally, while some methods—such as male and female condoms and oral contraceptive pills (Microgynon or Microlut)—were frequently reported, methods including cycle beads for the Standard Days Method, withdrawal, and other traditional methods were grouped into a single category due to low reporting frequency.

### Statistical analysis

The magnitude of second-trimester induced abortions was calculated (1) as the number of induced abortions occurring at 13 weeks gestational age or later per 100 women who reported induced abortions when presenting for PAC and (2) as the number of induced abortions occurring at 13 weeks gestational age or later per 100 PAC visits. 95% confidence intervals were calculated using the Wilson score interval with Yate’s continuity correction [25].

For the descriptive statistics, categorical independent variables were presented using counts and percentages, while continuous variables were described using medians and interquartile ranges (IQRs). Medians and IQRs were presented due to non-normal distributions of the continuous variables. Univariable analysis was employed to explore associations between predictor variables and the trimester of induced abortion. For age, number of children, and time (in weeks) of decision to abort, the Mann-Whitney U test was used. For the analysis of categorical variables, the chi-squared test was used when contingency table counts exceeded five. The Fisher’s Exact Test was employed when counts were five or less. For the univariable analysis, p-values below 0.050 were considered statistically significant.

Unadjusted logistic regression was conducted to assess relationships between second trimester induced abortion and each independent variable. Multivariable logistic regression analysis was then conducted with those variables found to be associated with the outcome at *p* < 0.100 in the unadjusted analysis. The multivariable analysis particularly focused on investigating the potentially mediating effects of two independent variables—previous attempts at induced abortion and delay in decision to abort—on the other factors found to be statistically significant in the unadjusted analysis. Statistical mediation was assessed using the Baron and Kenny method [26].

For the multivariable analyses to explore mediation, efforts were taken to address the low frequency of second trimester abortions in the dataset. Firstly, no more than two independent variables were considered at a time in the adjusted logistic regression analyses, with focus on factors hypothesized to be associated with second trimester abortions and potential mediating variables. Additionally, ahead of the analyses, multiple imputation was conducted with the entire analysis dataset (all variables found to be significant at *p* < 0.100 in the unadjusted analysis) to address missingness in independent variables of interest and reduce loss of information. Specifically, multiple imputation by chained equations (MICE) was used to iteratively impute each variable with missing values conditional on the other variables in the dataset. Ten imputed datasets were created based on 50 maximum number of iterations. Multiple regression analysis was then conducted with each of the imputed datasets and adjusted odds ratios, and 95% confidence intervals (95% CIs) were reported as pooled results across the ten datasets.

Among women whose abortions were classified as induced based on provider perspective, 17 did not consent to participate in patient PMS interviews; however, clinical data were available for the women via the provider PMS. Reasons for not answering the survey questions were most frequently due to a patient being discharged (and therefore not available for the survey) at a time when a data collector was not available. In some cases, women left the facility before being discharged (i.e., against medical advice) or they refused to consent to answering the questions. Data for these women are reflected in the calculation of the magnitude of second trimester abortions, but not in any other analyses.

### Ethics considerations

Prior to data collection for the overarching HFS and PMS, ethical approval was obtained from the University of Liberia-PIRE (now ACRE IRB) (Protocol #21-07-275), the Clinton Health Access Initiative’s (CHAI) Scientific and Ethical Review Committee (SERC), and the APHRC IRB. During the data collection period, informed verbal consent was obtained from all participants after providing comprehensive information about the study, including their right to withdraw. Participants who were minors seeking post-abortion care were treated as emancipated minors, and their consent was obtained directly. To ensure confidentiality given the sensitive nature of the topic, verbal consent was prioritized over written consent to avoid any identifying records. Strict measures were implemented to protect participant privacy and data confidentiality, including private data collection settings, password-protected and encrypted electronic data collection tools, secure data storage, and de-identification of data. Potential risks of emotional distress were acknowledged, and participants were informed about available counseling support. Participation was voluntary, and no incentives were provided, and the consent included the use of data for future secondary analysis. For the secondary analysis of the data presented, the researchers received deidentified datasets from the primary study.

## Results

A total of 431 women seeking post-abortion care (PAC) at 100 facilities across Liberia’s 15 counties participated in the patient PMS. Out of the 431 women, 246 (57.1%) were classified as having experienced a spontaneous abortion based on self-report and/or provider assessment, while 185 (42.9%) reported or were assessed as having induced abortions.

### Magnitude of second-trimester induced abortions

A total of 35 induced abortions were reported as occurring at 13 weeks gestation or after (i.e., in the second trimester). Out of all women coming to health facilities for PAC, regardless of whether their abortions were induced or spontaneous, 8.1% (95% CI: 5.9%, 11.0%) presented to the health facility in the second trimester and after an induced abortion. Among the 185 women seeking PAC after induced abortions, 19.0% (95% CI: 14.0%, 25.3%) had terminated their pregnancies during the second trimester. This means that out of every 100 women who induce abortion and present at facilities for PAC, 19—or nearly one-fifth—had terminated their pregnancies at 13 weeks gestation or later.

### Sociodemographic characteristics

The median age of women who induced abortion was 23 years old (IQR: 19–29 years, Range: 13–43 years) (Table 1). Nearly half of women seeking PAC for induced abortion were from urban settings (48.5%). A majority (59.6%) were living with their partner and were currently employed (67.7%). Religious affiliation reflects that of the Liberia population more broadly, with the large majority (90.4%) classifying themselves as Christians. Most (55.1%) had secondary schooling and above.

There were no statistically significant differences in terms of age, relationship status, religious affiliation, or employment status between women seeking PAC for abortions induced in the first versus second trimester (Table 1). The level of highest education attained was however significantly different between women seeking PAC for first-trimester induced abortions and women seeking PAC for second-trimester induced abortions (*p* = 0.045). Notably, a higher proportion of participants terminating in the second-trimester reported having no formal education or primary school level education as compared to those terminating in the first trimester (24.2% versus 14.2% for no formal education and 42.4% versus 25.4% for primary school, for second versus first trimester).

**Table 1:** Sociodemographic characteristics of women seeking post-abortion care for induced abortions

Characteristics	Overall (*n* = 184)	First Trimester (*n* = 149)*	Second Trimester (*n* = 35)*	*P*-value
**Age**				
** Median age (IQR)**,** in years**	23.0 (19.0–29.0)	23.5 (19.0-29.7)	21.0 (19.0–25.0)	0.102
**Setting of residence**				
** Urban**	81/167 (48.5)	68/134 (50.7)	13/33 (39.4)	0.252
** Semi-urban**	48/167 (28.7)	39/134 (29.1)	9/33 (27.3)	
** Rural**	38/167 (22.8)	27/134 (20.1)	11/33 (33.3)	
**Relationship status** (***n* = 18)**				
** Living with a man**	99/166 (59.6)	82/133 (61.7)	17/33 (51.5)	0.387
** Not living with a man**	67/166 (40.4)	51/133 (38.3)	16/33 (48.5)	
**Religious affiliation**				
** Christian**	151/167 (90.4)	123/134 (91.8)	28/33 (84.8)	0.317
** Muslim or Other**	16/167 (9.6)	11/134 (8.2)	5/33 (15.2)	
**Highest education level completed**				
** No formal education**	27/167 (16.2)	19/134 (14.2)	8/33 (24.2)	0.045
** Primary**	48/167 (28.7)	34/134 (25.4)	14/33 (42.4)	
** Secondary (Junior or senior ** **high school)**	76/167 (45.5)	67/134 (50.0)	9/33 (27.3)	
** Tertiary**	16/167 (9.6)	14/134 (10.4)	2/33 (6.1)	
**Employment status**				
** Not Working**	54/167 (32.3)	39/134 (29.1)	15/33 (45.5)	0.112
** Working**	113/167 (67.7)	95/134 (70.9)	18/33 (54.5)	

*For first-trimester patients (*n*=149), sociodemographic data were available for 134. For second-trimester patients (*n*=35), sociodemographic data were available for 33. Patients with missing PMS data had provider data; however, they either left before being discharged, were discharged at a time when the data collector was not available, or refused consent to the PMS survey.

### Reproductive history and context of current pregnancy

The difference in the number of children between those who induced abortions in the second trimester (Median: 3, IQR: 1–6) and those who induced in the first trimester (Median: 2, IQR: 0–9) was not statistically significant (Table 2). Similar proportions of women inducing in the first versus second trimester had experienced spontaneous abortion during a previous pregnancy (18.7% among first-trimester versus 18.2% among second-trimester patients) or had a history of unintended pregnancy (20.9% among first-trimester versus 21.2% among second-trimester patients). Relative to the 12.1% of women inducing in their second trimesters who reported a previous induced abortion, a higher but not statistically significant proportion of women inducing in the first trimester reported having induced abortion previously (17.9%).

**Table 2:** Reproductive history of women seeking post-abortion care for induced abortions

Characteristics	Overall (*n* = 184)	First Trimester (*n* = 149)	Second Trimester (*n* = 35)	*P*-value
Number of children				
** Median number (IQR)**	2.0 (0.0–9.0)	2.0 (0.0–9.0)	3.0 (1.0–6.0)	0.126
**History of spontaneous abortion**				
** Never**	136/167 (81.4)	109/134 (81.3)	27/33 (81.8)	1.000
** One or More**	31/167 (18.6)	25/134 (18.7)	6/33 (18.2)	
**History of induced abortion**				
** Never**	139/167 (83.2)	110/134 (82.1)	29/33 (87.9)	0.604
** One or More**	28/167 (16.8)	24/134 (17.9)	4/33 (12.1)	
**History of unintended pregnancy**				
** Never**	132/167 (79.0)	106/134 (79.1)	26/33 (78.8)	1.000
** One or More**	35/167 (21.0)	28/134 (20.9)	7/33 (21.2)	

The majority of women seeking PAC after induced abortion reported that their pregnancies were not intended, with 70.1% of women who induced in the first trimester and 51.5% of women who induced in the second trimester indicating that they had not wanted to get pregnant (Table 3). Around half of women reported being on family planning prior to their current pregnancy, with this figure being slightly lower among those who induced in the first trimester (63/134, 47.0%) versus second trimester (19/33, 57.6%). Among those who were on family planning, the methods were similar between women who induced abortion during the first versus second trimester. Most reported using an injectable; oral contraceptives and implants were also commonly reported.

**Table 3:** Context around the current pregnancy

Characteristics	Overall (*n* = 184)	First Trimester (*n* = 149)	Second Trimester (*n* = 35)	*P*-value
Pregnancy wantedness				
** Wanted to get pregnant**	56/167 (33.5)	40/134 (29.9)	16/33 (48.5)	0.068
** Did not want to get pregnant**	111/167 (66.5)	94/134 (70.1)	17/33 (51.5)	
**Using family planning prior to this pregnancy**				
** No**	85/167 (50.9)	71/134 (53.0)	14/33 (42.4)	0.372
** Yes**	82/167 (49.1)	63/134 (47.0)	19/33 (57.6)	
**Method of family planning**,** prior to this pregnancy**^a^				
** Oral Contraceptive**	14/82 (17.1)	10/63 (15.9)	4/19 (21.1)	0.692
** Condom**	1/82 (1.2)	1/63 (1.6)	0/19 (0.0)	
** Injectable**	50/82 (61.0)	38/63 (60.3)	12/19 (63.2)	
** Implant**	11/82 (13.4)	8/63 (12.7)	3/19 (15.8)	
** Traditional methods** ^b^	6/82 (7.3)	6/63 (9.5)	0/19 (0.0)	
**Time of decision to abort**				
** Median time (IQR)**,** in weeks**	5.0 (1.7–9.2)	4.0 (1.3-8.0)	12.0 (4.3–16.0)	< 0.001
**Previous attempt at inducing abortion** (***n* = 81)**				
** No**	60/103 (58.3)	53/81 (65.4)	7/22 (31.8)	0.010
** Yes**	43/103 (41.7)	28/81 (34.6)	15/22 (68.2)	

^a^Among those who indicated being on family planning prior to pregnancy that was aborted

^b^Traditional methods included the Standard Days Method, withdrawal, and other traditional methods which were combined into a single category due to low frequency of reporting

Overall, the median time between women realizing they were pregnant and deciding to abort was five weeks (IQR: 1.7–9.2 weeks) (Table 3). However, the distribution of the decision time in weeks significantly differed for women who ultimately terminated their pregnancies in the first versus second trimesters. Among women terminating in the first trimester, the median time for their decision to abort was four weeks (IQR: 1.3-8.0), while for women terminating in the second trimester, the median time increases substantially to 12 weeks (IQR: 4.3–16.0) (*p* < 0.001).

### Failed prior attempts at abortion

Women who induced abortion in the second trimester were significantly more likely to have made previous, unsuccessful attempts to terminate their pregnancies, compared to women who induced in the first trimester (*p* = 0.010) (Table 3). Nearly 70% of women who had second-trimester abortions (15/22, 68.2%) had tried more than once to end their pregnancies, while 34.6% of women who had first-trimester abortions (28/81) had previously tried to end their pregnancies.

Among the 43 women who tried more than once to induce abortion, their first unsuccessful attempts at inducing abortion occurred at a median 7.5 weeks gestation (IQR: 4.0–12.0). The initial attempts at inducing abortion were primarily carried out using herbal or home remedies (15/43, 34.9%), misoprostol/cytotec (14/43, 32.6%), and unspecified tablets referred to by patients as “other” (9/43, 20.9%). Additionally, dilation and curettage (D&C) was reported by two participants (2/43, 4.7%), while three participants (3/43, 7.0%) used other methods that could not be classified per the other categories based on the description provided by the patients. Herbal or home remedies, misoprostol/cytotec, and “other” pills remained the three most common methods for subsequent attempts, including the final attempt that resulted in the abortion. Among the 15 patients who utilized misoprostol/cytotec in their final (and successful) efforts to terminate prior to presenting at the facility, the majority (11/15, 73.3%) acquired it from a pharmacy, while the remaining one-quarter of patients (4/15, 26.7%) sourced it from markets, street vendors, health facilities, and other channels. After their initial unsuccessful attempts at abortion, 24 individuals (55.8%) reported that the later attempt involved a method obtained from a non-healthcare worker; whereas 19 individuals (44.2%) indicated a healthcare worker as the provider.

### Factors associated with increased odds of second-trimester abortion

The unadjusted logistic regression analyses identified four variables significantly associated with timing of induced abortion at *p* < 0.100. These included highest education level, whether or not the pregnancy was intended, timing of decision to abort, and having made prior attempts to terminate the same pregnancy (Table 4). The odds of second-trimester abortion was significantly higher for women with primary school-level or no education, relative to women with secondary school-level or higher education (OR = 3.1; 95% CI: 1.4-7.0), and for women who intended to get pregnant, relative to women who had not wanted to get pregnant (OR = 2.2; 95% CI: 1.0-4.8). Moreover, delay in decision-making around termination of the pregnancy was associated with higher odds of second-trimester abortion. Specifically, for each additional week it took for a woman to decide to terminate her pregnancy, the odds of a second-trimester abortion increased by 20% (OR: 1.2, 95% CI: 1.1–1.4). Previous attempts at inducing abortion for the current pregnancy were also significantly associated with our outcome; women with a previous (but unsuccessful) attempt at inducing abortion had 4.1 times the odds of inducing in the second trimester compared to those patients without such a history (95% CI: 1.5, 11.7).

After adjusting for previous attempts at termination, whether the pregnancy was intended or not was no longer significantly associated with the timing of the abortion, although the magnitude of the odds ratios did not change (Table 4). This suggested there may be some degree of mediation by previous attempted abortion in the pathway between the factor and delay to the second trimester. More investigation is warranted with larger sample sizes. Education remained statistically significantly associated with abortion in the second trimester, after adjustment for previous attempts at termination, although the magnitude of the effect was attenuated. In the multiple logistic regression adjusting for previous attempts at abortion, the odds of a second-trimester abortion among women with primary school education or less was 2.5 times the odds of a second-trimester abortion among women with secondary or tertiary education (95% CI: 1.0-5.9).

After adjusting for timing (in weeks) of decision to abort, neither education level nor whether the pregnancy was intended was statistically significantly associated with second-trimester abortion (Table 4). This suggests that lower educational attainment may be associated with a longer decision-making process which then leads to abortion later in pregnancy; likewise, decision-making for abortion of intended pregnancies may have taken longer, thereby leading to second-trimester abortions.

In the adjusted analysis, both increasing time to make a decision to abort and having made previous attempts at aborting the pregnancy remained statistically significantly associated with increased odds of second-trimester abortion (Table 4).

**Table 4:** Results of logistic regression analysis to explore independent factors associated with timing of abortion (first versus second trimester) and potential mediating effects

			Analysis 1: Adjusting for Previous Abortion Attempts		Analysis 2: Adjusting for Time to Decision	
Variables	Unadjusted OR (95% CI)	*P*	Adjusted OR (95% CI)	*P*	Adjusted OR (95% CI)	*P*
Education						
** No formal education or primary school education**	3.1 (1.4, 7.0)	0.006	2.5 (1.0, 5.9)	0.046	1.6 (0.6, 4.3)	0.375
** Secondary or tertiary education**	Ref					
**Intention to get pregnant prior to conception**						
** Wanted to get pregnant**	2.2 (1.0, 4.8)	0.045	2.2 (0.9, 5.3)	0.076	1.6 (0.6, 4.5)	0.360
** Did not want to get pregnant**	Ref					
**Time of decision to abort**						
** Timing**,** in weeks**	1.2 (1.1, 1.4)	< 0.001	1.3 (1.1, 1.5)	0.004		
**Previous attempt at inducing**						
** Yes**	4.1 (1.5, 11.7)	0.006			3.9 (1.3, 11.6)	0.017
** No**	Ref					

## Discussion

Our results find that nearly half of women presenting to sampled facilities for PAC either self-reported or were assessed as having induced their abortions. Further, our results suggest that nearly one-fifth of induced abortions, among women seeking PAC, occurred in the second trimester. The analysis shows, unsurprisingly, that people who delayed decision-making on whether to abort or who had previous unsuccessful abortion attempts were more likely to terminate in the second trimester. The analysis also demonstrates how these factors can help to explain the pathways between other commonly reported factors–notably, pregnancy intentions and education level–and second-trimester abortions.

Several studies have established the association between lower education levels and second-trimester abortions [27–30], as well as how pregnancy intention is related to decisions to abort [3]. However, investigating mediation processes can provide both statistical and conceptual evidence [31] about where more investigation and understanding are needed or where intervention may be warranted. Such evidence is particularly critical in Liberia, which has limited evidence on abortion and which is currently considering a change in the legal status of abortion, and thus potentially expanding access to safe abortion care, with ongoing revision of its Public Health Law.

In our study, a high proportion of second-trimester abortions were among women with intended pregnancies; yet, the direct effect of intention to get pregnant on terminating in the second trimester became insignificant after adjusting for time of decision to abort and for previous unsuccessful attempts to terminate the pregnancy. While it is noted that full mediation cannot be concluded based on changes in the p-values alone, the effects of pregnancy intention on timing of the decision to abort makes sense conceptually in the context of Liberia. In particular, some women who initially intended to carry their pregnancies to term may have experienced changes in personal, social, or economic circumstances that led them to reconsider. These shifts in the wantedness of the pregnancy can result in delayed decision-making or more complicated decision processes around abortion and abortion methods. Additionally, hesitation due to cultural or societal expectations may lead these women to attempt less effective methods of self-termination, further contributing to delays to successful termination and increasing the likelihood of second-trimester procedures.

The direct effect of lower education level on abortion in the second trimester was mediated by time of decision to abort, with women attaining no formal or primary school education deciding to terminate later in their pregnancies than women with higher educational attainment. This finding is consistent with prior studies that reported significant associations between delayed timing of abortion and lower education levels. This may reflect not only differences in decision-making processes but also disparities in access to information and services that ultimately influence individual agency. Women with lower education levels may have less awareness of early pregnancy signs, or the risks associated with later procedures. They may also face difficulties identifying where to go for abortion services, identifying the most effective methods, securing funds needed, or overcoming social constraints. For instance, although our study did not explicitly measure cultural beliefs about gestational age and abortion, qualitative studies [10, 11, 32] suggest that some women perceive second-trimester abortions as more feasible or likely to succeed. For example, some people believed that the pregnancy must “grow enough” to be terminated, pointing to cultural or experiential understandings that may inadvertently delay care-seeking. Also, the pursuit for social safety (referring to the preservation of identity and social relationships, avoidance of shame and stigmatization) may drive women towards using mixed methods in multiple attempts that are unsuccessful further protracting the abortion process [9]. These challenges can result in delayed recognition of pregnancy, postponed decision-making, and delayed access to abortion care—all of which increase the likelihood of second-trimester termination.

Access to safe abortion care is known to reduce maternal mortality [33]. The impact is likely higher in settings where second-trimester abortions are more prevalent. At the global level, estimates for the prevalence of second-trimester abortions range from 10 to 15% [7]. Studies specific to the SSA region report higher local estimates. For instance, a study conducted in the Amhara region of Ethiopia reported the magnitude of second-trimester abortions at 19.2% of all induced abortions [10]. Another study among women receiving safe abortion services at public hospitals in southern Ethiopia reported a slightly higher prevalence of 23% [34]. Other estimates have been markedly higher–from 30% of women presenting for abortion care in Debre Markos, Ethiopia [35] to 39% of women presenting for PAC for induced abortions in a nationally representative sample in Kenya [36]. Our findings around the magnitude of second-trimester abortion among women seeking PAC are similar to other estimates in the SSA region with legally restrictive abortion laws. This corroborates our analysis, despite its limited sample size, and reflects that higher-risk, second-trimester abortions may be occurring frequently in Liberia. It also emphasizes the importance of understanding and mitigating factors that delay abortions, such as decision-making and earlier term but ineffective, and mostly unsafe induced abortion methods and procedures.

Access to and use of drugs for medication abortion (misoprostol, or combined misoprostol and mifepristone) has increased in low-income settings where abortion is restricted [37, 38]. These drugs provide safe and highly effective options for self-induced abortion without engagement with the formal healthcare system. In Liberia, a wide range of abortion methods was reported—including misoprostol, herbal treatments, and other pills which patients could not identify. Decisions to use accessible and in some cases less safe methods is consistent with findings from Kenya and Benin, where women reported prioritizing discretion over safety when attempting to terminate a pregnancy [9]. Misoprostol can serve as a resource to women seeking both safe and discrete methods of pregnancy termination, and expanded access to misoprostol could help women access safe abortion earlier in pregnancy.

Importantly, over half of the women in our study seeking second-trimester post-abortion care reported using family planning methods prior to their current pregnancy. While this indicates a degree of engagement with contraceptive services, it also suggests gaps in quality of contraceptive counseling, method choice, and usage. Strengthening contraceptive services and education is therefore crucial—not only to prevent unintended pregnancies but also to reduce the risk of unsafe or later term abortions regardless of pregnancy intention. As efforts to expand legal access to safe abortion care progress, ensuring contraceptive needs are met among women wishing to avoid pregnancy must remain a central strategy in improving reproductive health outcomes [25, 37–39].

Delayed abortions in legally restrictive settings result from complex and myriad factors that influence decisions and behavior [39]. In parallel to the social and psychological costs of unsafe abortion in legally restrictive settings [40], the economic costs associated with post-abortion care complications resulting from unsafe abortion can be significant [41, 42]. One study in Zambia estimated that post-abortion care following an unsafe abortion has 2.5 times the cost implications of safe abortion care [42]. Thus, in resource-constrained settings with many competing health priorities, improved access to safe abortion results in fewer complications [40], reducing maternal morbidity and mortality and rendering resources more available for other less preventable health emergencies.

### Limitations

The present study was a secondary data analysis. As such, the factors investigated for associations with timing of abortion were limited to those variables for which data were collected. While structural factors, for instance, could not be assessed, the analysis did account for many of the individual-level factors that had been shown to influence delays in abortion in other settings. Likewise, since the primary outcome of interest to the original research was not related to gestational age at abortion, the sample size for the present study was not sufficient for a multiple regression analysis beyond the mediation analysis adjusting for two independent variables at a time. Primary data collection with sample size calculations related to second-trimester abortion as the primary outcome of interest could allow for additional analyses, including more rigorous exploration of causal pathways, in future research. Furthermore, as with other research on abortion and similarly sensitive topics in legally restrictive settings, self-report data may reflect social desirability bias or intentional inaccuracy due to fear of social or legal consequences of accurate responses. When possible, self-report data was compared with and/or supplemented with clinical assessment data. Future research in Liberia can draw from established methods or consider locally appropriate novel methods to overcome such limitations and further expand the evidence base around abortions in general and higher risk second trimester abortions. Finally, our analysis is limited to people seeking PAC, and does not purport to make any conclusions about the population-level incidence of second-trimester induced abortion.

## Conclusions

The findings reveal that nearly one-fifth of abortions among women seeking post-abortion care in Liberia occurred in the second trimester. The predominant association between previous unsuccessful attempts at abortion and delay to the second-trimester before a successful abortion underscores the importance of interventions to expand access to safe and effective abortion methods, particularly medication abortion. Efforts should be directed towards ensuring access to comprehensive reproductive health education, especially targeting women with lower educational attainment, to empower them with knowledge on safe and timely abortion practices. Additionally, addressing the issue of unintended pregnancies through improved access to contraceptives and family planning services, including counseling on effective use, can play a crucial role in reducing the prevalence of second-trimester abortions. While the findings align with similar studies in low-income settings, variations observed in different regions highlight the importance of undertaking contextually relevant research that considers local contexts, laws, and health systems. A rigorous evidence base for Liberia is increasingly critical as the country navigates potential changes to its legal landscape around sexual and reproductive health services.

## Data Availability

All data and materials are available on request from the corresponding author. Also, according to our institutional policies (APHRC - the organization hosting the datasets), all deidentified datasets will be publicly available on the APHRC microdata portal after 3 years (https://aphrc.org/microdata-portal/).

## References

[1] Bankole A, Remez L, Owolabi O, Philbin J, Williams P. From unsafe to safe abortion in Sub-Saharan Africa: Slow but steady progress, New York: Guttmacher Institute, 2020, https://www.guttmacher.org/report/from-unsafe-to-safe-abortion-in-subsaharan-africa.

[2] Ganatra B, Gerdts C, Rossier C, Johnson BR Jr, Tunçalp Ö, Assifi A, et al. Global, regional, and subregional classification of abortions by safety, 2010-14: estimates from a bayesian hierarchical model. Lancet. 2017;390:2372–81. 28964589 10.1016/S0140-6736(17)31794-4 PMC5711001

[3] Bearak J, Popinchalk A, Ganatra B, Moller A-B, Tunçalp Ö, Beavin C, et al. Unintended pregnancy and abortion by income, region, and the legal status of abortion: estimates from a comprehensive model for 1990–2019. Lancet Glob Health. 2020;8:e1152–61. 32710833 10.1016/S2214-109X(20)30315-6

[4] Abortion in Sub-Saharan Africa. Guttmacher Institute. 2020. https://www.guttmacher.org/fact-sheet/abortion-subsaharan-africa. Accessed 14 Feb 2024.

[5] Waithaka A, Evidence on Abortion Status and Trends Across Sub-Saharan Africa.: Guttmacher and AFIDEP Session at FIGO 2020 Congress - African Institute for Development Policy - AFIDEP %. African Institute for Development Policy - AFIDEP. 2020. https://www.afidep.org/unintended-pregnancy-and-unsafe-abortion-in-sub-saharan-africa-new-report-by-guttmacher-institute/. Accessed 14 Feb 2024.

[6] Rehnström Loi U, Gemzell-Danielsson K, Faxelid E, Klingberg-Allvin M. Health care providers’ perceptions of and attitudes towards induced abortions in sub-Saharan Africa and Southeast asia: a systematic literature review of qualitative and quantitative data. BMC Public Health. 2015;15:139. 25886459 10.1186/s12889-015-1502-2 PMC4335425

[7] Shah I, Ahman E. Unsafe abortion: global and regional incidence, trends, consequences, and challenges. J Obstet Gynaecol Can. 2009;31:1149–58. 20085681

[8] Grossman D, Constant D, Lince N, Alblas M, Blanchard K, Harries J. Surgical and medical second trimester abortion in South africa: a cross-sectional study. BMC Health Serv Res. 2011;11:224. 21929811 10.1186/1472-6963-11-224 PMC3196698

[9] Omondi GA, Both J, Ouedraogo R, Kimemia G, Juma K. I wasn’t sure it would work. I was just trying: an ethnographic study on the choice of abortion methods among young women in Kilifi county, kenya, and Atlantique department, Benin. Reprod Health. 2023;20:181. 38057868 10.1186/s12978-023-01720-x PMC10701931

[10] Mulat A, Bayu H, Mellie H, Alemu A. Induced second trimester abortion and associated factors in Amhara region referral hospitals. Biomed Res Int. 2015;2015:256534. 25918704 10.1155/2015/256534 PMC4396136

[11] Harries J, Orner P, Gabriel M, Mitchell E. Delays in seeking an abortion until the second trimester: a qualitative study in South Africa. Reprod Health. 2007;4:7. 17883835 10.1186/1742-4755-4-7 PMC2039724

[12] Drey EA, Foster DG, Jackson RA, Lee SJ, Cardenas LH, Darney PD. Risk factors associated with presenting for abortion in the second trimester. Obstet Gynecol. 2006;107:128–35. 16394050 10.1097/01.AOG.0000189095.32382.d0

[13] Gallo MF, Nghia NC. Real life is different: a qualitative study of why women delay abortion until the second trimester in Vietnam. Soc Sci Med. 2007;64:1812–22. 17355899 10.1016/j.socscimed.2007.02.005

[14] Varkey SJ. Abortion services in South africa: available yet not accessible to all. Int Fam Plan Perspect. 2000;26:87–8.

[15] Harrison A, Montgomery ET, Lurie M, Wilkinson D. Barriers to implementing South africa’s termination of pregnancy act in rural kwazulu/natal. Health Policy Plan. 2000;15:424–31. 11124246 10.1093/heapol/15.4.424

[16] Jewkes RK, Gumede T, Westaway MS, Dickson K, Brown H, Rees H. Why are women still aborting outside designated facilities in metropolitan South africa?? BJOG. 2005;112:1236–42. 16101602 10.1111/j.1471-0528.2005.00697.x

[17] Ahlbach C, Puri MC, Daniel S, Rocca CH, Karki S, Foster DG. Predictors of prior unsuccessful pharmacy abortion attempts among women presenting for abortion in government certified clinics in Nepal. Int J Gynaecol Obstet. 2022;159:160–5. 35152426 10.1002/ijgo.14141 PMC9372217

[18] Obure VA, Juma K, Athero S, Donzo V, Conteh-Khali N, Ouedraogo R, et al. Sometimes you have knowledge but lack the equipment to save a life: perspectives on health system barriers to post-abortion care in Liberia and Sierra Leone. Arch Public Health. 2024;82:220. 39574170 10.1186/s13690-024-01446-7 PMC11580692

[19] Ushie BA, Giorgio M, Juma K, Donzo V, Philbin J, Lu L et al. Estimating unintended pregnancy and induced abortion in liberia: A nationally representative cross-sectional survey, 11 July 2024, PREPRINT (Version 1) available at Research Square [https://doi.org/10.21203/rs.3.rs-4595818/v1]

[20] Ndyanabangi B, Livingstone M, Gobeh W, Logan GG, Atukunda L. Unsafe abortion - A risk factor for maternal mortality in Liberia - An analysis of the characteristics of unsafe abortion clients and risk factors for maternal morbidity and mortality. Afr J Reprod Health. 2021;25:43–50. 37585819 10.29063/ajrh2021/v25i6.5

[21] Odunvbun WO, Kollie JT. The burden of surgical complications from unsafe abortion treated at the John F. Kennedy maternity center (JFKMC), monrovia, Liberia. Malawi Med J. 2022;34:43–8. 37265831 10.4314/mmj.v34i1.8 PMC10230577

[22] Liberia Institute of Statistics and Geo-Information Services-LISGIS. Ministry of Health-MOH, ICF. Liberia Demographic and Health Survey 2019-20. 2021

[23] Moseson H, Massaquoi M, Dehlendorf C, Bawo L, Dahn B, Zolia Y, et al. Reducing under-reporting of stigmatized health events using the list experiment: results from a randomized, population-based study of abortion in Liberia. Int J Epidemiol. 2015;44:1951–8. 26342584 10.1093/ije/dyv174 PMC5156336

[24] Government of Liberia, Liberia Institute of Statistics and Geo-Information Services (LISGIS). United Nations Population Fund (UNFPA). Liberia Population and Housing Census 2022. 2024.

[25] Newcombe RG. Two-sided confidence intervals for the single proportion: comparison of seven methods. Stat Med. 1998;17:857–72. 9595616 10.1002/(sici)1097-0258(19980430)17:8<857::aid-sim777>3.0.co;2-e

[26] Baron RM, Kenny DA. The moderator-mediator variable distinction in social psychological research: conceptual, strategic, and statistical considerations. J Pers Soc Psychol. 1986;51:1173–82. 3806354 10.1037//0022-3514.51.6.1173

[27] Rocca CH, Puri M, Dulal B, Bajracharya L, Harper CC, Blum M, et al. Unsafe abortion after legalisation in nepal: a cross-sectional study of women presenting to hospitals. BJOG. 2013;120:1075–83. 23574112 10.1111/1471-0528.12242

[28] Abebe M, Mersha A, Degefa N, Gebremeskel F, Kefelew E, Molla W. Determinants of induced abortion among women received maternal health care services in public hospitals of Arba minch and Wolayita Sodo town, Southern ethiopia: unmatched case-control study. BMC Womens Health. 2022;22:107. 35397584 10.1186/s12905-022-01695-0 PMC8994190

[29] Jones RK, Kost K. Underreporting of induced and spontaneous abortion in the united states: an analysis of the 2002 National survey of family growth. Stud Fam Plann. 2007;38:187–97. 17933292 10.1111/j.1728-4465.2007.00130.x

[30] Upadhyay UD, Cartwright AF, Grossman D. Barriers to abortion care and incidence of attempted self-managed abortion among individuals searching Google for abortion care: A National prospective study. Contraception. 2022;106:49–56. 34560051 10.1016/j.contraception.2021.09.009

[31] Agler R, De Boeck P. On the interpretation and use of mediation: multiple perspectives on mediation analysis. Front Psychol. 2017;8:1984. 29187828 10.3389/fpsyg.2017.01984 PMC5694788

[32] Lie MLS, Robson SC, May CR. Experiences of abortion: a narrative review of qualitative studies. BMC Health Serv Res. 2008;8:150. 18637178 10.1186/1472-6963-8-150 PMC2488341

[33] Ganatra B, Faundes A. Role of birth spacing, family planning services, safe abortion services and post-abortion care in reducing maternal mortality. Best Pract Res Clin Obstet Gynaecol. 2016;36:145–55. 27640082 10.1016/j.bpobgyn.2016.07.008

[34] Abebe M, Mersha A, Degefa N, Molla W, Wudneh A. Magnitude of second-trimester-induced abortion and associated factors among women who received abortion service at public hospitals of Arba minch and Wolayita Sodo towns, Southern ethiopia: A cross-sectional study. Front Glob Womens Health. 2022;3:969310. 36312870 10.3389/fgwh.2022.969310 PMC9614144

[35] Tesfaye B, Tewabe M, Ferede A, Dawson A. Induced second trimester abortion and associated factors at Debre Markos referral hospital: Cross-Sectional study. Womens Health. 2020;16:1745506520929546. 10.1177/1745506520929546 PMC7315676 32578513

[36] Ushie BA, Izugbara CO, Mutua MM, Kabiru CW. Timing of abortion among adolescent and young women presenting for post-abortion care in kenya: a cross-sectional analysis of nationally-representative data. BMC Womens Health. 2018;18:41. 29452587 10.1186/s12905-018-0521-4 PMC5816362

[37] Stillman M, Owolabi O, Fatusi AO, Akinyemi AI, Berry AL, Erinfolami TP, et al. Women’s self-reported experiences using Misoprostol obtained from drug sellers: a prospective cohort study in Lagos state, Nigeria. BMJ Open. 2020;10:e034670. 32376752 10.1136/bmjopen-2019-034670 PMC7223139

[38] Moore AM, Blades N, Ortiz J, Whitehead H, Villarreal C. What does informal access to Misoprostol in Colombia look like? A mystery client methodology in Bogotá and the coffee Axis. BMJ Sex Reprod Health. 2020;46:294–300. 32624479 10.1136/bmjsrh-2019-200572 PMC7569367

[39] Hinson L, Bhatti AM, Sebany M, Bell SO, Steinhaus M, Twose C, et al. How, when and where? A systematic review on abortion decision making in legally restricted settings in sub-Saharan africa, Latin america, and the Caribbean. BMC Womens Health. 2022;22:415. 36217197 10.1186/s12905-022-01962-0 PMC9552475

[40] Haddad LB, Nour NM. Unsafe abortion: unnecessary maternal mortality. Rev Obstet Gynecol. 2009;2:122–6. 19609407 PMC2709326

[41] Henshaw SK, Adewole I, Singh S, Bankole A, Oye-Adeniran B, Hussain R. Severity and cost of unsafe abortion complications treated in Nigerian hospitals. Int Fam Plan Perspect. 2008;34:40–50. 18440916 10.1363/ifpp.34.140.08

[42] Parmar D, Leone T, Coast E, Murray SF, Hukin E, Vwalika B. Cost of abortions in zambia: A comparison of safe abortion and post abortion care. Glob Public Health. 2017;12:236–49. 26708223 10.1080/17441692.2015.1123747

